# Successful Evolution of Atrial Septal Defect Closure in an Elderly Patient With Complex Cardiovascular Disease

**DOI:** 10.1016/j.case.2024.05.006

**Published:** 2024-06-17

**Authors:** Raul Miranda-Segura, Jose Carlos Armendariz-Ferrari, Pavel Martinez-Dominguez, Maria Jose Santa Ana-Bayona, Enrique C. Guerra, Nilda Espinola-Zavaleta

**Affiliations:** a Department of Nuclear Cardiology, National Institute of Cardiology Ignacio Chavez, Mexico City, Mexico; b Department of Echocardiography, National Hospital Hipolito Unanue, Lima, Peru; c Department of Echocardiography, ABC Medical Center, Mexico City, Mexico

**Keywords:** Atrial septal defect, Congenital heart disease, Echocardiography, Atrial fibrillation

## Abstract

• ASD closure in elderly patients improves RV remodeling.
• ASD closure is feasible in older patients with arrhythmias and pulmonary hypertension.
• Clinical and hemodynamic status is improved after ASD closure.

## Graphical abstract

**Figure:**
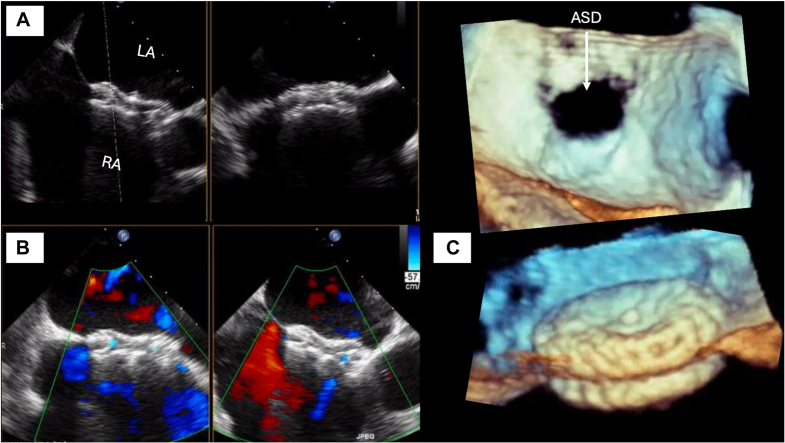

## Introduction

Recent advances in early diagnosis, medical interventions, and surgical treatment have significantly contributed to the growing population of adult patients with congenital heart disease (CHD).^1^ These individuals are susceptible to cardiovascular risk factors and may potentially develop conditions such as coronary artery disease (CAD), acute myocardial infarction, and other atherosclerotic cardiovascular diseases, including cerebrovascular disease and heart failure.^2^^,^^3^

Atrial septal defect (ASD) is the most common CHD diagnosed in adulthood, accounting for 25% to 30% of diagnoses.^4^ Device closure is the treatment of choice when feasible and is associated with improved survival and low mortality.^5^ Surgical closure of the ASD before the age of 25 years decreases complications during adulthood.^6^ However, in the elderly, it is also beneficial and is therefore considered as a treatment option.^7^

Echocardiography with current modalities remains the first-line noninvasive imaging technique for ASDs. This technique gives information on the shape, size, and location of defects, allows evaluation of right ventricular (RV) chamber size and function and estimation of RV pressures, and serves as a guide during interventional closure procedures.^8^

We present the successful evolution of a 72-year-old patient with CAD and CHD after percutaneous coronary intervention (PCI) and ASD percutaneous closure.

## Case Presentation

A 72-year-old woman with a history of hypertension, diabetes, dyslipidemia, obesity (body mass index of 36 kg/m^2^), chronic obstructive pulmonary disease, and atrial fibrillation was evaluated in the emergency department for acute chest pain and tachyarrhythmia. Five years prior, the patient had been diagnosed with ASD; in light of the high risk for surgical treatment, the patient opted not to undergo ASD closure.

**Figure 1:**
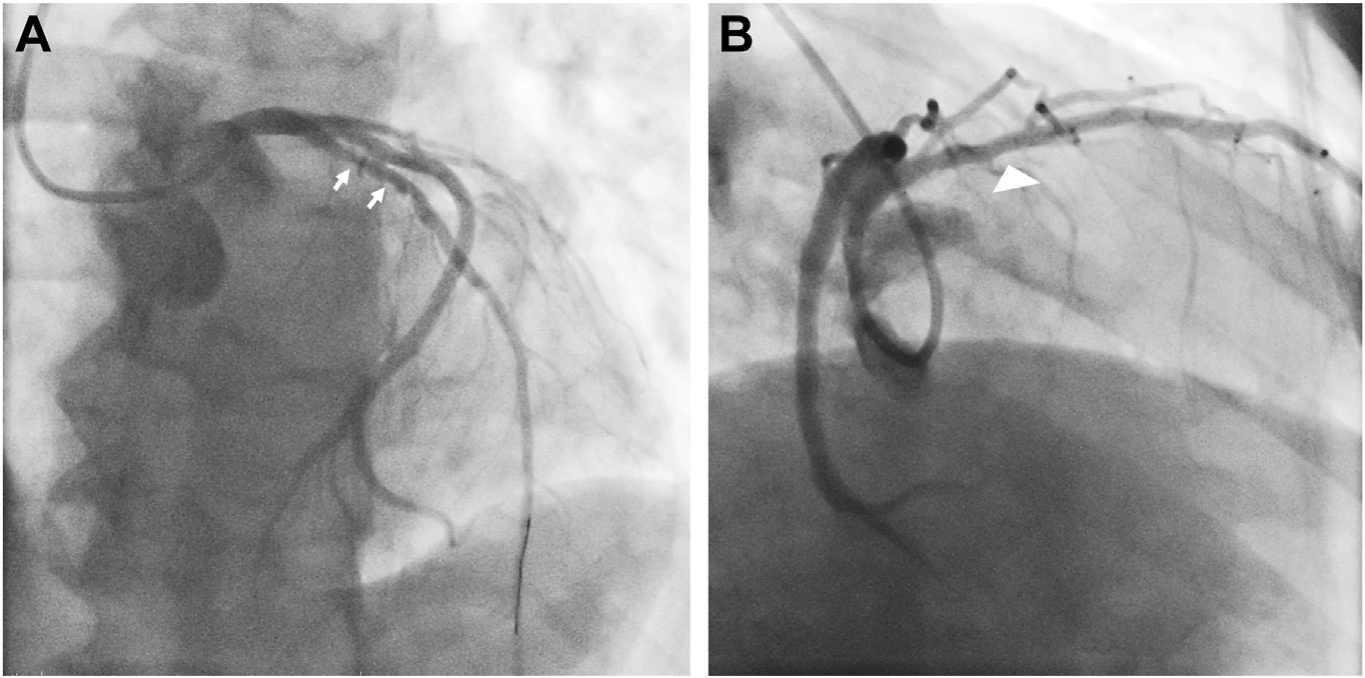
Selective invasive coronary angiography demonstrates an obstructive lesion of the left anterior descending artery (90%) in its proximal segment (*arrows*, **A**) which was successfully treated with PCI (*arrowhead*, **B**).

**Figure 2:**
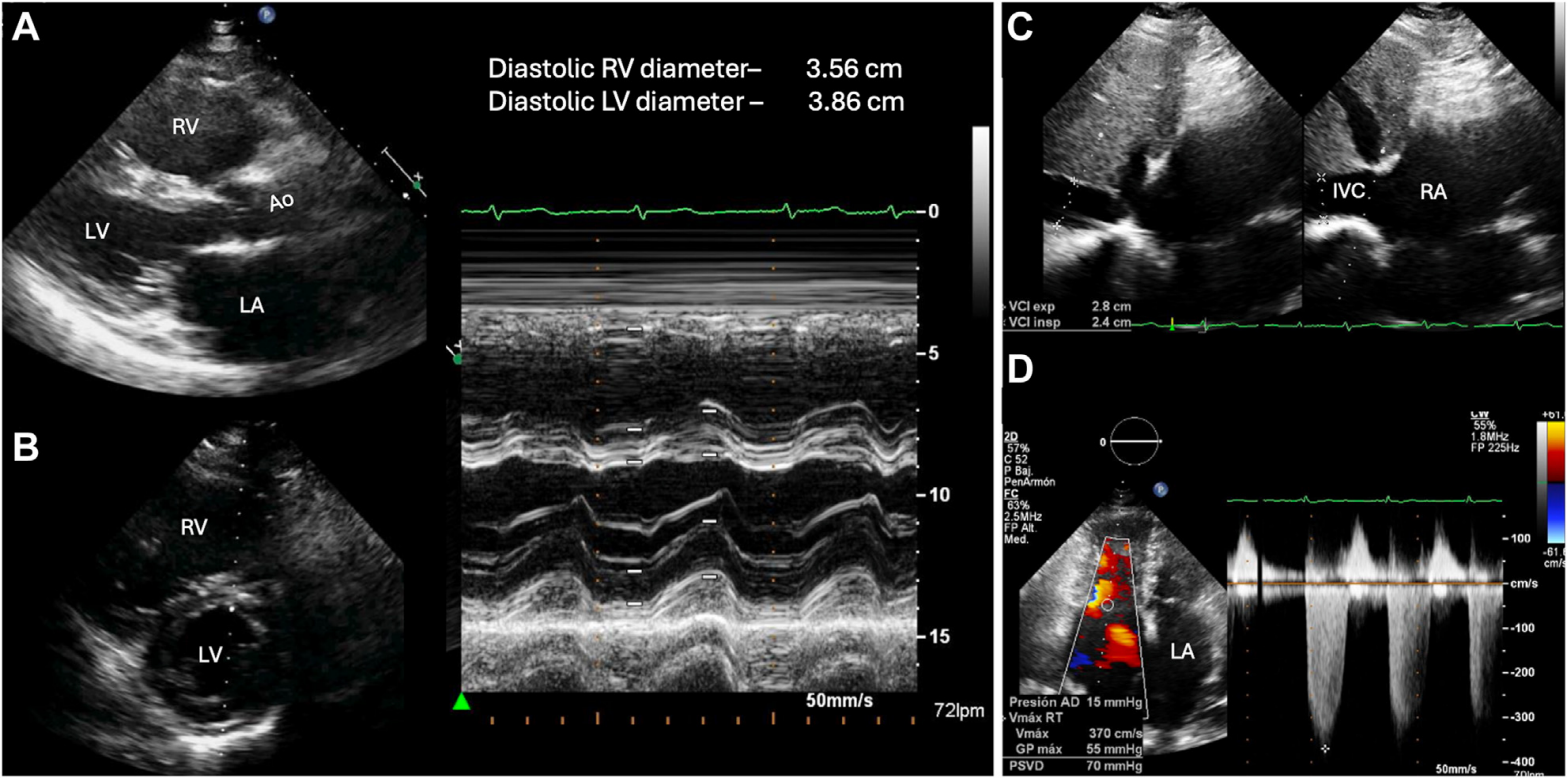
Two-dimensional TTE-guided M-mode, parasternal long-axis **(A)** and short-axis **(B)** views, demonstrate RV dilation and paradoxical movement of the IVS. Subcostal view of the inferior vena cava **(C)** during inspiration (*left*) demonstrates less than 50% inspiratory collapse. Right ventricle–focused apical 4-chamber view **(D)** with color-flow Doppler-guided continuous-wave Doppler, reveals a peak TR velocity of 3.7 m/sec and estimated RV systolic pressure of 70 mm Hg. *Ao*, Aorta; *IVC*, inferior vena cava; *LA*, left atrium; *LV*, left ventricle; *RA*, right atrium; *RV*, right ventricle.

The electrocardiogram showed atrial fibrillation with rapid ventricular response, right bundle branch block, and left anterior fascicular block, prompting further evaluation. The coronary angiogram demonstrated an obstructive lesion of 90% in the proximal segment of the left anterior descending coronary artery, which was resolved by PCI with stent implantation (Figure 1). After PCI, the patient remained in New York Heart Association class II to III, with poor functional capacity during the Naughton protocol stress test despite medical management with antihypertensive therapy and adequate heart rate control. The case was reevaluated, and transthoracic echocardiography (TTE) showed dilation of the right heart with paradoxical motion of the interventricular septum (IVS) and a peak tricuspid regurgitation (TR) velocity of 3.7 m/sec indicative of a high probability of pulmonary hypertension (PH; Figure 2), left-to-right shunt, preserved biventricular systolic function with left ventricular (LV) ejection fraction of 72% by the Simpson method, tricuspid annular plane systolic excursion of 25 mm, and RV ejection fraction of 52% by three-dimensional TTE. Hemodynamic data were calculated as follows: *Qp:Qs* of 2.3:1, pulmonary vascular resistance of 3 Woods units, mean pulmonary artery pressure of 45 mm Hg, and pulmonary capillary wedge pressure of 13 mm Hg.

**Figure 3:**
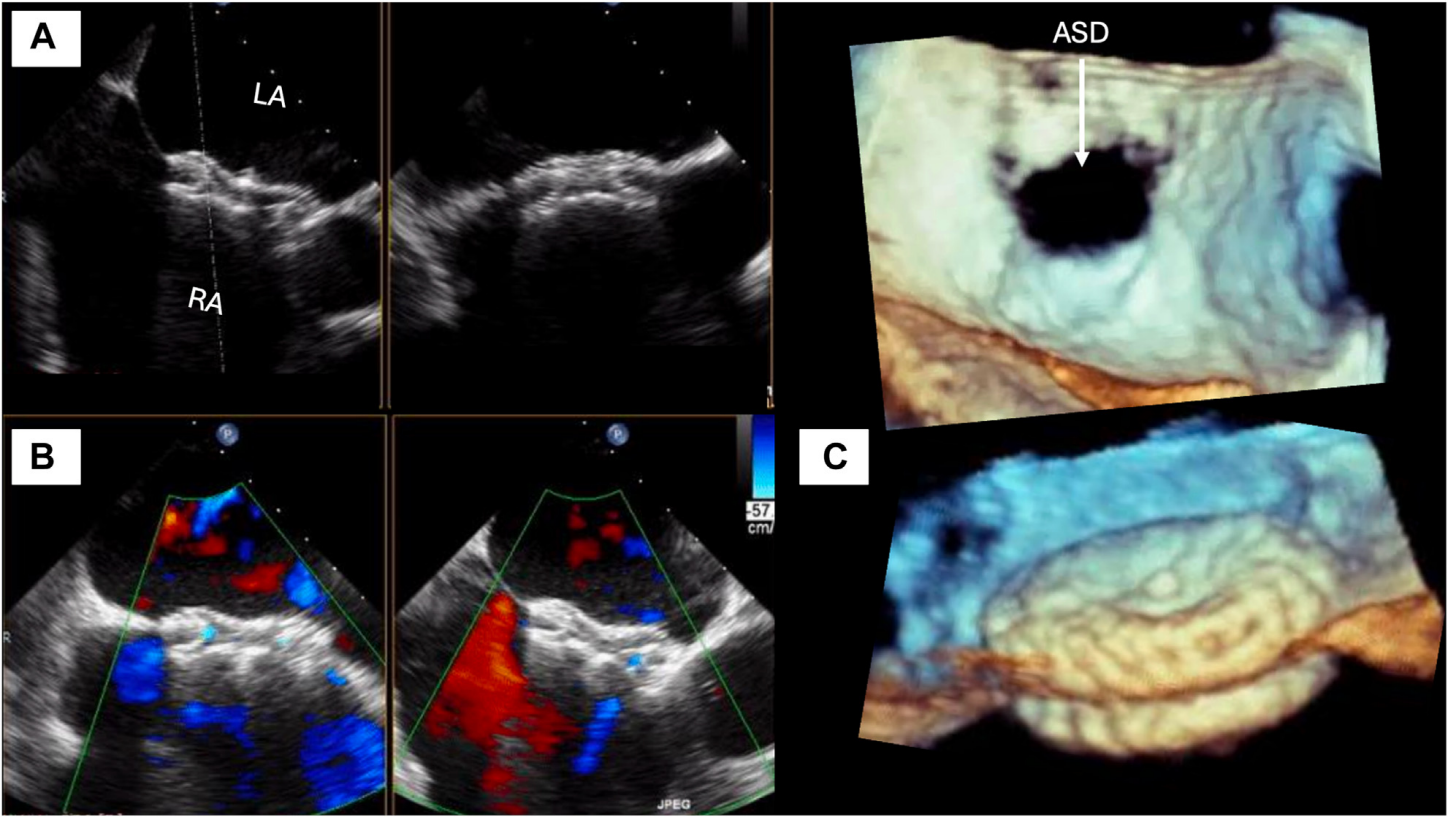
Two-dimensional transesophageal echocardiography, orthogonal biplane atrial septal views without **(A)** and with **(B)** color-flow Doppler, demonstrates the normal appearing, well-positioned septal occluder device without residual shunting. Three-dimensional transesophageal echocardiography, volume-rendered display of the ostium secundum ASD **(C)** from the perspective of the RA at baseline (*top*) and a zoomed, oblique view after repair with a well-positioned septal occluder device (*bottom*). *LA*, Left atrium; *RA*, right atrium.

**Figure 4:**
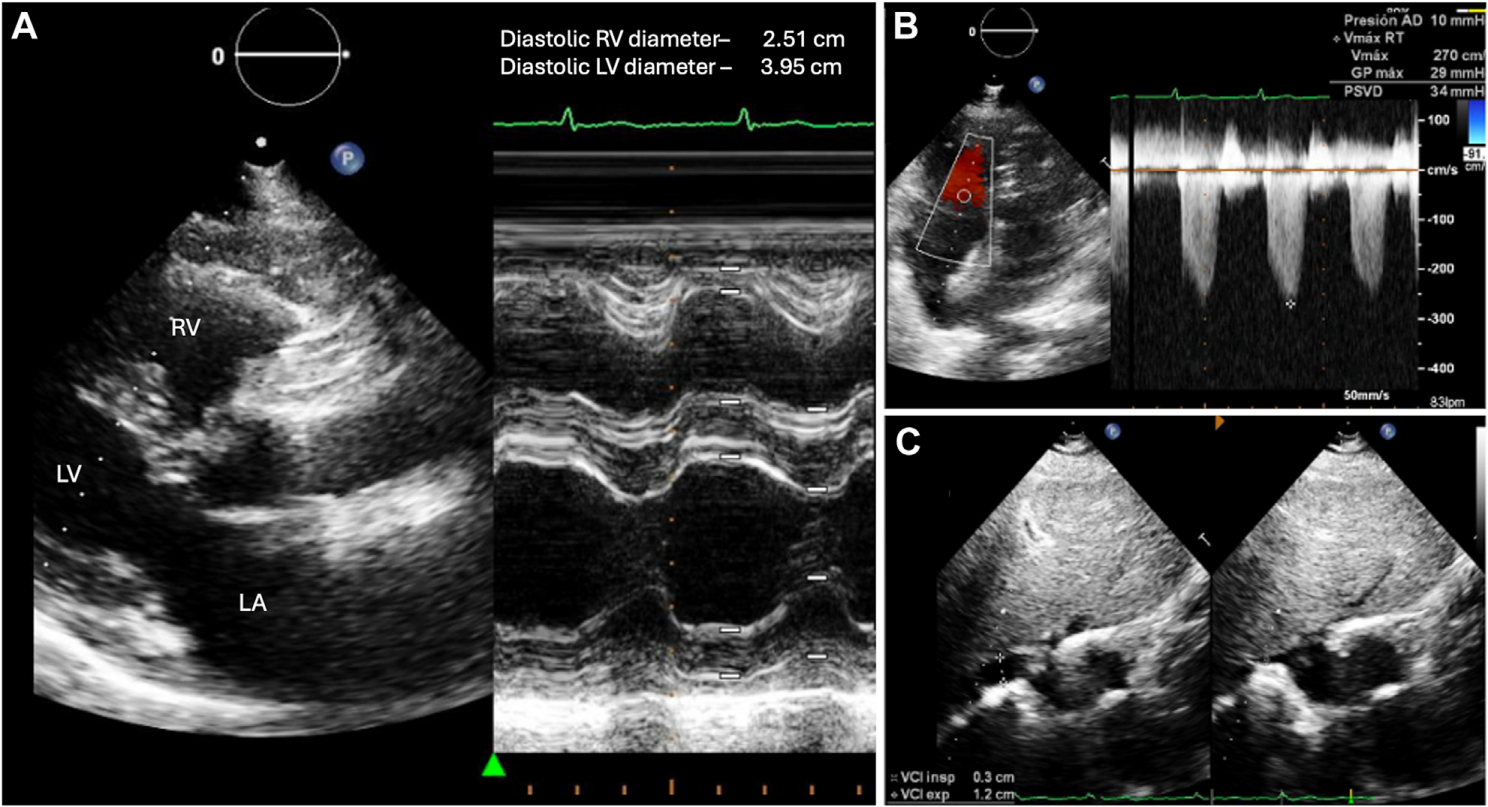
Two-dimensional TTE-guided M-mode, parasternal long-axis view **(A)** 24 months post-ASD closure, demonstrates normal RV size and interventricular septal motion. Right ventricle–focused apical 4-chamber view **(B)** with color-flow Doppler-guided continuous-wave Doppler reveals a peak TR velocity of 2.7 m/sec and normal estimated RV systolic pressure. Subcostal view **(C)** demonstrates a normal diameter of the inferior vena cava with normal inspiratory collapse. *LA*, Left atrium; *LV*, left ventricle; *RV*, right ventricle.

The transesophageal echocardiography showed an ostium secundum ASD (25.54 mm), which was successfully closed using a septal occluder device (Figure 3, Videos 1-3). The patient was discharged 4 days after the procedure with a functional New York Heart Association class I, receiving medical treatment with aspirin, clopidogrel, and warfarin. Follow-up with TTE at 12 months demonstrated a well implanted septal occluder device, flattened IVS, severe TR (vena contracta of 8.4 mm), and a peak TR velocity of 3.1 m/sec with an intermediate probability of PH. Subsequent follow-up with TTE at 24 months evidenced normalized motion of the IVS and a peak TR velocity of 2.7 m/sec with a low probability of PH with a calculated systolic pulmonary artery pressure of 34 mm Hg (Figure 4). Serial TTE revealed marked improvement in right heart size and function (Table 1, Video 4).

**Table 1:** Baseline right heart echocardiographic parameters and follow-up

Parameter	Baseline	12-month follow-up	24-month follow-up
Right atrial volume, mL/m^2^	54	52	50
RV basal diameter, mm	35.6	34.3	25.1
Tricuspid annular plane systolic excursion, mm	16	19	23
S’ wave, cm/sec	8.8	10.4	12.5

The patient remained in atrial fibrillation with normal ventricular rate at follow-up. Currently, the heart rhythm is atrial fibrillation with normal ventricular response.

## Discussion

Atrial septal defect is the most common congenital heart lesion in adults. There are 4 types of ASDs: coronary sinus (<1%), sinus venosus (10%), ostium primum (15%), and ostium secundum. The ostium secundum accounts for 75% of ASDs and is a true defect of the atrial septum occurring in the region of the fossa ovalis.^7^

Several studies have shown a causal association between CHD and the development of CAD. This association primarily arises from the presence of age-related risk factors in adults with CHD. Although conventional cardiovascular risk factors are more prevalent in older patients, younger patients still encounter a high risk of CAD. This elevated risk can be attributed to factors related to CHD, such as impaired anatomy and function of the coronary circulation.^1^

The treatment of ASD in elderly patients may be complicated by the abovementioned risk factors. The presence of comorbidities represents a challenge in treating ASD in this population. Over a third of patients exhibit systemic hypertension and other systemic diseases, such as diabetes mellitus or history of stroke. Additionally, cardiac comorbidities, such as PH, ventricular dysfunction, atrial arrhythmia, and ischemic heart disease, are known to further complicate transcatheter closure.^9^

A recent study has evaluated the potential benefits of transcatheter ASD closure on the elderly. This analysis demonstrated the favorable effects in RV size, which had a beneficial impact on the patient’s functional capacity, as well as increased LV size and reduced pulmonary pressures, with no modifications in the incidence of arrythmias after the procedure. These benefits can be achieved even in patients over 60 years of age and decrease all-cause mortality after correction of ASD.^5^

Transcatheter ASD closure is a safe procedure even in elderly patients; however, late correction is associated with major complications after the procedure, such as arrhythmias, stroke, PH, ventricular remodeling, valvular heart disease, and heart failure.^10^ Among the arrhythmias, atrial flutter and atrial fibrillation are the most common. In patients >60 years of age, 20% experience atrial flutter and 50% atrial fibrillation.^11^ These are the result of the continuous right atrial dilation and stretch due to ASD. However, in elderly patients, consequent sudden cardiac death is not common.^12^

Previous studies have shown that advanced age at the time of closure and a history of atrial arrhythmias before closure are strong predictors of atrial arrhythmias after ASD closure.^13^ However, the history of arrhythmias does not constitute a contraindication or reduce the benefits of ASD closure. Several studies have demonstrated that patients with permanent atrial fibrillation who have successfully undergone transcatheter closure have achieved improvements in cardiac remodeling and functional class after the procedure, even patients over 60 years of age.^14^ Furthermore, transcatheter ASD closure remains as an effective treatment for older patients with PH, because it prevents progression and improves long-term survival, even in presence of concomitant valvular heart disease.^15^

Several studies have described the efficacy of transcatheter ASD closure in elderly patients; however, some failed to achieve the expected benefit. Therefore, a temporary closure of ASD by percutaneous balloon inflation has recently become relevant, because it can assess any abnormalities in LV systolic or diastolic function that may predict the likelihood of acute congestive heart failure after the procedure in elderly patients.^16^

In the 24-month follow-up of our patient who underwent percutaneous closure of ASD, the RV remodeling occurred with normalization of the contractility and the pulmonary pressure.

## Conclusion

Early diagnosis and prompt treatment in adult patients with CHD are associated with successful outcomes and improved survival. However, ASD closure in the elderly is associated with a significant clinical and hemodynamic improvement, even when there are significant associated comorbidities, as in our patient, who also developed CAD. Therefore, ASD closure can be considered as a first-line therapeutic option in the elderly.

## Ethics Statement

The authors declare that the work described has been carried out in accordance with The Code of Ethics of the World Medical Association (Declaration of Helsinki) for experiments involving humans.

## Consent Statement

Complete written informed consent was obtained from the patient (or appropriate parent, guardian, or power of attorney) for the publication of this study and accompanying images.

## Funding Statement

The authors declare that this report did not receive any specific grant from funding agencies in the public, commercial, or not-for-profit sectors.

## Disclosure Statement

The authors report no conflict of interest.

## Supplementary data

Video 1 Three-dimensional transesophageal echocardiogram, focused volume-rendered serial displays of the ASD from the right atrial perspective, demonstrates the catheter positioned within the ASD, followed by confirmation of the septal occluder device securely positioned within the interatrial septum.

Video 2 Two-dimensional transesophageal echocardiogram, bicaval (65°) and biplane orthogonal long-axis views, confirms the optimal position of the septal occluder device within the interatrial septum.

Video 3 Two-dimensional transesophageal echocardiogram, bicaval (65°) and biplane orthogonal long-axis views with color-flow Doppler, showing the septal occluder device securely implanted within the interatrial septum, with no evidence of residual shunt.

Video 4 Two-dimensional transesophageal echocardiogram, RV-focused apical 4-chamber view with color-flow Doppler preprocedure **(A)** and without color-flow Doppler after repair **(B)**, serves to reveal marked remodeling of the right heart at 24 months of follow-up. The ASD is seen at baseline **(A),** and the occluder device is seen on follow-up **(B)**.
